# Research on accurate perception and control system of fertilization amount for corn fertilization planter

**DOI:** 10.3389/fpls.2022.1074945

**Published:** 2022-11-25

**Authors:** Bo Wang, Yafei Wang, Hui Wang, Hanping Mao, Liming Zhou

**Affiliations:** ^1^ College of Agricultural Engineering, Jiangsu University, Zhenjiang, China; ^2^ Chinese Academy of Agricultural Mechanization Sciences, Beijing, China

**Keywords:** precision fertilization, fertilizer flow sensor, intelligent control, corn fertilizer planter, feedback PID control system

## Abstract

At present, there are excessive fertilizer use and poor uniformity of fertilizer discharge in corn fertilizer planter. The key difficulty is that accurate perception and control of fertilizer amount has not been solved. Aiming at the above problems, a set of accurate perception and control system applied to corn fertilization planter was studied. According to the difference in dielectric properties between fertilizer and air, a sensor for online detection of fertilizer amount based on capacitance method was designed. And the relationship model of mass flow rate for N, P, K fertilizer and capacitance output was established. In order to reduce the influence of pulsation on fertilization flow, a high-precision fertilizer flow control system for fertilization planter based on the fertilizer flow feedback and PID control method was designed. The validated results showed that the maximum measurement error between the relationship model and capacitance output was 3.75%. As the temperature rises from room temperature to 55°C, the differential capacitance change rate of the sensor was less than 3%. The steady-state error of fertilizer discharge was less than 2%. The field experiment of the accurate perception and control system for corn fertilization amount show that the electric drive fertilization system has good consistency, the maximum and average variation coefficient of fertilization were 3.74%, 1.6%, respectively, and the variable control accuracy was greater than 97%. The control accuracy of the grain spacing control by electric drive seed metering was 98%. Therefore, the precision fertilization control system in this study can realize high-precision and on-demand fertilization. It is of great significance to realize the intelligence and precision for corn fertilization planter.

## 1 Introduction

Corn is the main food crop in China, and it has basically realized mechanized planting, management and harvest (Darwin et al., 2021; Wang et al., 2021). However, there is still the problem of excessive application of chemical fertilizer when applying fertilizer to corn (Yang et al., 2016). Precise fertilization planter technology and equipment are the key support for solving the current over-application of agricultural production materials fertilizers in China (Zhao et al., 2010). At present, developed countries such as Europe and the United States have widely used precision sowing equipment. In order to improve the efficiency and quality of domestic corn fertilization and sowing, China urgently needs to research an independent precise fertilizer planter with intelligent decision-making and control technology suitable for national conditions (Yuan et al., 2018).

In the research of variable fertilization based on the working prescription map, the “SOILECTION” fertilization system produced by Open Ag-chen Instrument Equipment Co., Ltd. in the United States can be used for dry fertilization or liquid fertilization. It adjusts the application amounts of nitrogen fertilizer, phosphate fertilizer and potash fertilizer according to the fertilization prescription map (Poncet et al., 2018). Karayel et al. (Karayel and Özmerzi, 2002) used Ag-Chem’s commercialized product FALCON control system to carry out variable fertilization experiments of two liquid fertilizers on an 8-row fertilizer applicator, and carried out static calibration, dynamic response test and field test of the system, and obtained better test results. Trimble Company, John Deere Company and Case Company in the United States, AMAZONE Company in Germany and the All-Russian Institute of Agricultural Mechanization in Russia have respectively developed variable fertilizer applicators and variable fertilizer control systems, and the French AMASAT fertilizer spreading variable control system is also widely used in Various types of centrifugal fertilizer spreaders (Hakansson et al., 2011). Japan’s Kubota, Jingseki, Yanmar and other companies have carried out a lot of research work on rice lateral deep fertilization equipment, and have developed matching lateral deep fertilization equipment for their respective rice transplanters. Domestic units such as The Soil-Machine-Plant Key Laboratory of the Ministry of Agriculture of China (He et al., 2019), China Agricultural University (Cai et al., 2011), Inner Mongolia Agricultural University (Wen et al., 2014), Shanghai Jiao Tong University (Yuan et al., 2010) and Nanjing Agricultural University (Ding et al., 2021) have also carried out development and research of variable fertilizer applicators to support precision production are carried out. Nielsen et al. (Nielse et al., 2018) used the single-chip microcomputer to control the speed of the fertilizer shaft of the fertilizer applicator, and realized a variable fertilizer operation system with 2 rows and a continuously adjustable fertilizer rate of 200-500 kg/ha. Zhao etc. (Zhao et al., 2015) used 89C51 single-chip to control the electronically controlled hydraulic drive system, drive the fertilizer discharge device, and realize variable fertilization of the seeder. Closed-loop control was adopted in the system, which effectively improves the dynamic response characteristics and steady-state performance of the system. Further research showed that the system can comprehensively process information such as GPS, GIS, sensor information and decision-making data. When the working speed of the stepper motor is in the range of 33-91r/min, the average error of fertilizer discharge by the fertilizer applicator is 4.22% (Huang et al., 2015). Li etc. (Li et al., 2016) used AgGPSl70 and Mid-Tech TASC6100 variable operation controller, equipped with domestic tractors and fertilizer spreaders, and trial-produced a set of variable fertilizer operation system suitable for spreading fluidity and granular fertilizers. Fu etc. (Fu et al., 2018) designed a self-propelled variable fertilization machine with high ground clearance in paddy fields, which can fertilize at a variable speed according to the machine, and the error of fertilization amount is 5%.

Although the above methods can realize variable fertilization, it is limited by the fact that the real-time fertilizer flow rate is not obtained, so that the real-time fertilizer flow is not controlled. Moreover, the fertilizer discharge pulsation of the fertilizer discharge device is obvious, and the uniformity of fertilizer discharge is poor, thus affecting the fertilization accuracy. Fertilizer quantity detection is the key to implement automatic control of precise fertilization, and it is also the basis for judging the quality level of fertilization and sowing operation. At present, photoelectric detection sensors are widely used in fertilization detection parameters (Liu and Yi, 2019; Chen et al., 2022). However, there are problems such as environmental light, temperature interference and field dust influence, and there is still a lack of effective detection means of fertilizer application amount (Chen et al., 2022). Dielectric constant is used to describe the degree of electric field weakening caused by a dielectric, and it is the main macroscopic physical quantity that comprehensively reflects the internal polarization of a dielectric. In this study, according to the difference in dielectric properties between fertilizer and air, a sensor for online detection of fertilizer amount based on capacitance method was designed to reduce the influence of environment. In order to ensure the stability of fertilization and the accuracy of fertilization amount, the measured granular fertilizer flow value was used as the feedback value of the control system to adjust the fertilizer flow in real time. Based on PID control method, a high-precision fertilizer flow control system for fertilizer seeder was designed. It is of great significance for realizing the intelligence and precision of corn fertilization sowing.

## 2 Development of online detection sensor for fertilizer application based on capacitance method

### 2.1 Capacitance model and structure of online detection sensor for fertilization amount

When the fertilizer passes through the capacitor electrode, the equivalent dielectric constant of the medium between the capacitor electrode is *Ɛ*. There is:


(1) $$Ɛ=\frac{V_{1}}{V}{Ɛ}_{1}+\frac{V_{2}}{V}{Ɛ}_{2}$$

Where, *V*_1_ is used to represent the volume occupied by fertilizer, and $V_{1}=\frac{m_{1}}{\rho_{1}}$ , m^3^; *m*_1_ is used to represent the fertilizer quality in the sensor detection range, kg; *ρ*_1_ is used to represent the fertilizer density, kg/m^3^; *V*_2_ is used to represent the volume occupied by air, m^3^; *V* is used to represent the total volume of the detection range between the capacitor electrode for the capacitance sensor, and *V*=*V*_1_ + *V*_2_, m^3^; *Ɛ*_1_ is used to represent the dielectric constant of fertilizer, F/m; *Ɛ_2_* is used to represent the dielectric constant of air, F/m.

When no fertilizer passes through, the sensor capacitance value is:


(2) $$C_{0}={Ɛ}_{2}\frac{s}{d}$$

where, *s* is used to represent the capacitor electrode area, m^2^; *d* is used to represent the capacitor electrode spacing.

When the fertilizer passes through the capacitance sensor, the sensor capacitance value is:


(3) $$C=\frac{s{Ɛ}_{2}}{d}+\frac{s({Ɛ}_{1}-{Ɛ}_{2})}{\rho_{1}Vd}m_{1}$$

Thus, when fertilizer passes through the capacitance sensor, the capacitance sensor value will change, and the change amount of capacitance value is:


(4) $$C-C_{0}=\frac{s({Ɛ}_{1}-{Ɛ}_{2})}{\rho_{1}Vd}m_{1}$$

where, *C*-*C*_0_is used to represent the change in capacitance of the fertilizer as it passes through the sensor, pF.

It can be known from formula (4) that when fertilizer passes through the sensor, the output capacitance of the sensor will change, and the amount of change is linearly related to the quality of fertilizer. When the fertilizer passes, the capacitance value returns to the initial state. Therefore, by collecting the output capacitance of the sensor in real time, the online detection of fertilizer mass flow can be realized through the dynamic change of the capacitance sensor.

In addition, when the fertilizer pipe is blocked, the fertilizer in the sensor detection range rapidly accumulates, the quality of the fertilizer increases significantly, and the output capacitance of the sensor also increases sharply, thus realizing the monitoring and alarming of the blockage fault for the fertilizer discharge pipe.

**Figure 1:**
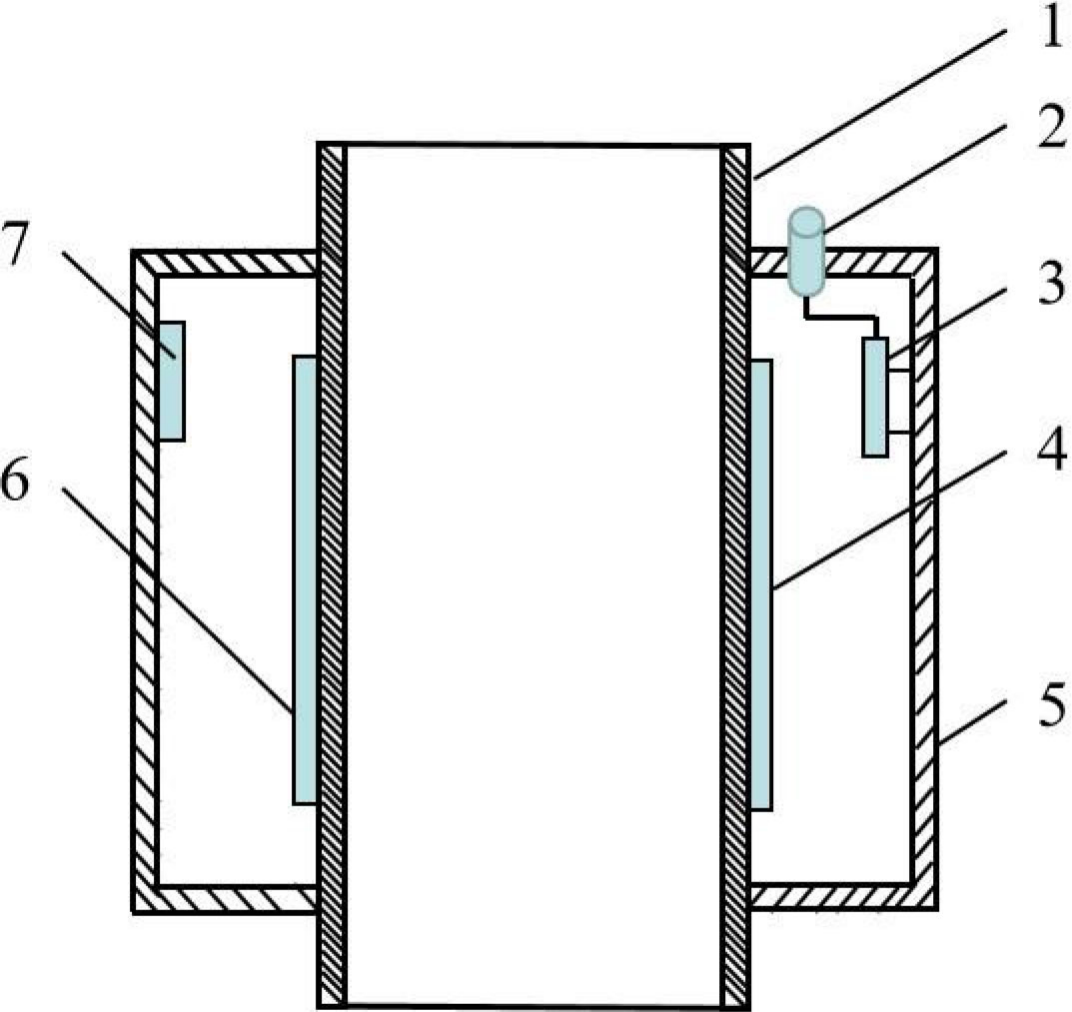
Structure of fertilization amount online sensor based on capacitance. 1. Fertilizer pipe; 2. Signal output connector; 3. Conditioning circuit board; 4. Detection capacitance electrode 1; 5. Shield shell; 6. Detection capacitance electrode 2; 7. Reference capacitance electrode.

The capacitance fertilizer flow sensor (structure as shown in **Figure 1**) is mainly composed of fertilizer discharge pipe, detecting capacitance electrode, reference capacitance electrode, signal conditioning circuit, shielding shell and so on. The fertilizer discharge pipe was made of PVC pipe, and the detection capacitance electrode was pasted on the periphery of the PVC fertilizer discharge pipe with 302 glue. There was a metal shielding shell outside the fertilizer discharge pipe, and a reference capacitance electrode was attached to the inside of the shielding shell. The electrode was made of 0.05 mm thickness copper foil tape, forming the fertilizer discharge detection pipe with inner and outer ring structure. According to the principle of capacitance induction, the larger the size of the electrode, the larger the basic capacitance of the sensor, the larger the area of induction, and the enhanced sensitivity to the medium between the capacitance electrode. In this study, the length of capacitance electrode 1 and 2 were designed to be 90 mm, and the width of capacitance electrode 1 and 2 were designed to be 43 mm. Capacitance electrode 3 is the reference capacitor. The length of capacitor electrode 3 was designed to be 40 mm, and the width of capacitor electrode 3 was designed to be 20 mm. Capacitance electrode 1 and 2 constitute the detection capacitance sensor, and capacitance electrode 2 and 3 constitute the reference capacitance sensor, thus constituting the capacitance sensor with a differential input structure. In this study, the basic capacitance of the designed detection sensor and reference capacitor was 4.2 pF. At the same time, a metal shielding shell was designed outside the whole detection pipe to improve the anti-interference performance. The signal conditioning circuit was located in the shield shell to reduce the influence of parasitic capacitance, improve detection sensitivity and base capacitance stability.

Generally, the external environment temperature and measured object’s water content will affect the output of the capacitance sensor. The fertilizer planter uses granular compound fertilizer. Chinese national standard stipulates (GB/T15063-2009) that the moisture content of compound fertilizer should be less than or equal to 5%, and the fertilizer moisture absorption is small, the moisture content change is small when applying, so the influence of moisture can’t be considered.

In order to eliminate the influence of environmental temperature change on the measurement results as much as possible, the ceramic capacitance with the same value of the sensor detection capacitance was used as the reference capacitance. The difference between the detected capacitance of the sensor and the reference capacitance was taken as the calculation result, so as to eliminate the possible influence of environmental temperature on capacitance detection to the greatest extent. According to the differential capacitance structure in **Figure 1**, the differential capacitance can be obtained as follows:


(5) $$C_{\text{d}}=C-C_{0}-C_{r}$$

where, *C*_d_ is used to represent the differential capacitance, pF; *C*_r_is used to represent the reference capacitance, pF.

Since the detection capacitance and the reference capacitance are in the same environment temperature, the influence caused by temperature variation can be eliminated theoretically through the differential capacitance calculation formula.

### 2.2 Relationship between fertilizer quality and capacitance sensor output

Based on the principle of capacitance sensing to realize the detection of fertilization flow, the sensor detection model of fertilization flow-capacitance information was studied. And the relationship model between the mass flow of nitrogen, phosphorus and potassium fertilizers and the capacitance response was established. The online detection sensor of fertilizer amount was installed at a proper position below the fertilizer outlet, and a material receiving box was placed at the fertilizer outlet of the online detection sensor, and the fertilizer application amount was measured by the mass method. The rotation speed of fertilizer discharge shaft was set as 20 r/min, and the change of fertilizer quality was realized by controlling the rotation time of fertilizer discharge shaft. After each fertilization, the electronic balance (SL4001, Shanghai Minqiao Electronic Instrument Factory, measuring range of 4 000 ± 0.1 g) was used to weigh the fertilizer quality in the receiving box, and the accumulated capacitance value of the difference between the capacitance sensor and the reference capacitance sensor was recorded at the same time. One calibration test was conducted for each fertilizer. Matlab R2016b was used to process the calibration test data, and the relationship model between the fertilizer mass flow and accumulated capacitance per unit time was obtained by linear fitting.


(6) $$Q=a\sum C_{\text{d}}+b$$

where, *Q* is used to represent the real-time fertilizer flow, g/s; *a* is used to represent the sensitivity of the relationship between capacitance value and fertilizer quality; *b* is used to represent the intercept of the relationship between capacitance value and fertilizer quality.

The linear fitting results are shown in **Table 1**. Due to the dielectric constant of different fertilizers being different, different fertilizers of the same quality have different capacitance output responses, so the scaling coefficients of the models are also different.

**Table 1:** Mass flow and capacitance response models of three fertilizers.

Fertilizer	Fit relation	Decisive factor
Nitrogen fertilizer (Urea)	*Q*=103.39 Σ*C*_d_ + 36.983	0.9898
Phosphate fertilizer (Super Phosphate)	*Q*=80.41 Σ*C*_d_ + 54.616	0.9889
Potash fertilizer (Potassium Chloride)	*Q*=34.46 Σ*C*_d_ - 11.909	0.9933

In order to verify the accuracy of the response model, a verification test of the detection performance of the fertilization sensor was carried out. The system detection effect was tested when the rotation speed of the drainage shaft was 25r/min and 35r/min respectively. and the test results are shown in **Table 2**. The results shown that the detection accuracy of capacitance fertilizer sensor for different fertilizer quality was more than 96%.

**Table 2:** Validation results of regression model.

Fertilizer	Test serial number	Actual mass (g)	Measurement result (g)	Error(%)
Nitrogen fertilizer	1	207.3	212.746	2.63
	2	305.2	295.458	-3.19
Phosphate fertilizer	1	319.6	311.918	-2.40
	2	405.7	416.447	2.65
Potash fertilizer	1	135.2	132.816	-1.76
	2	267.5	277.541	3.75

The fertilizer flow sensor was placed in the electric heating drying oven, and the internal temperature of the drying oven was gradually adjusted from room temperature (20°C) to 55°C with a gradient of 5°C. After each adjustment, the detection capacitance value *C*, reference capacitance *C_r_* and temperature *T* of the sensor were recorded after the display temperature was stable for 1min. When there was no fertilizer in the measuring device, the detection capacitance *C* of the sensor increases significantly with the increase of the environmental temperature. At 55°C, the capacitance value has changed by more than 7% compared with that at 20°C. After adopting the differential input structure, when there was no fertilizer in the sensor, the temperature rises, and the differential capacitance of the sensor was basically zero with no obvious fluctuation. After fertilizer sample was inserted into the sensor, the change rate of differential capacitance of the sensor remained within 3% as the temperature increased. It can be seen that the differential input structure can effectively weaken the interference of the environmental temperature change on the capacitance sensor.

## 3 Development of control system for corn fertilization planter

### 3.1 The unit composition of the corn fertilization planter detection and control system

**Figure 2:**
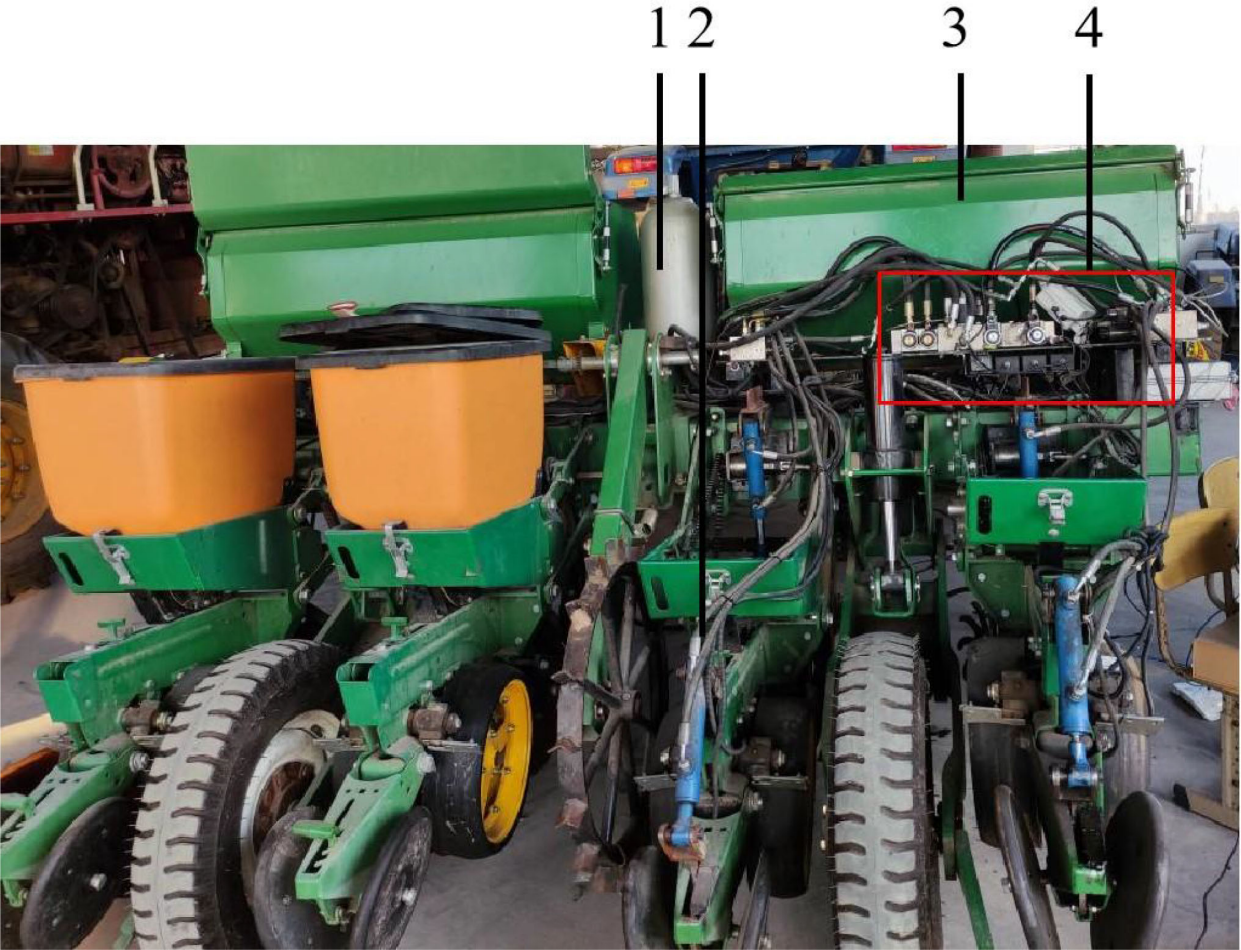
Fertilization measurement and control system of corn planter. 1.Energy accumulator 2. Electro-hydraulic regulating monomer 3. Fertilizer box 4. Hydraulic valve group.

The detection and control system for corn fertilization planter mainly consists of a vehicle terminal, a motor control unit, sowing and fertilizing drive motor unit, as shown in **Figure 2**.

Terminal application IPCA-7010 industrial vehicle computer integrates storage, communication, display, input and output modules, which has good compatibility and expansibility, is easy to operate and maintain, and can better meet the precision agricultural field operation process. The terminal integrates GPS positioning module, CAN bus module, DTU unit and so on.

The BG 45×15 SI integrated DC motor was used as the drive source of the seed metering shaft and the fertilizer discharging shaft. The motor has an integrated drive circuit inside to realize the dynamic adjustment of the motor speed with a flow analog voltage signal, and the signal range is 0-10V. The output power of the motor is 52.5W, the working voltage is DC12V, and the maximum speed is 3080 r/min. At the same time, it was also equipped with a PLG52 planetary reducer with a reduction ratio of 50. After the on-board terminal passes the decision, it sends the control command to the seeding drive motor and the fertilization drive motor through the CAN bus to drive the seed metering shaft and the fertilization axis to rotate.

The K8516 CAN bus analog output module was used as motor control unit, and its main technical parameters are as follows: 4 output signals, 12-bit DA resolution, output signal range 0-10V and power supply voltage DC9-24V. It communicates with the vehicle terminal through the CAN bus interface.

### 3.2 Design of corn precision fertilization control system

At present, the research of fertilization control system is mainly to accurately adjust the speed of fertilizer discharge motor according to the forward speed of the machine and the measured speed of the fertilizer discharge motor. However, since the outer groove wheel fertilizer discharge is mostly used in corn planters, its mechanical structure determines that it has pulsating characteristics during fertilizer discharge, and there is no linear relationship between the fertilizer discharge amount and the rotation speed (An et al., 2019). Therefore, based on the fertilizer discharge motor fertilizer quantity control with rotational speed feedback cannot achieve high-precision and precise fertilizer discharge. In order to solve this problem, the optimal design of corn precision fertilization control system was carried out. The capacitance fertilizer flow sensor was installed on the fertilizer discharge pipe to detect the real-time fertilizer discharge amount, and the speed of the fertilizer discharge motor was adjusted based on the real-time flow feedback, so as to realize the precise control of the fertilizer discharge amount.

#### 3.2.1 Modeling of fertilizer flow control system

The capacitance fertilizer discharge flow sensor was installed on the fertilizer discharge pipe to detect the real-time fertilizer discharge. On the basis of obtaining the fertilizer flow, the fertilizer amount per unit area can be further obtained according to operating width and forward speed of the fertilizer machine, and the speed of the fertilizer discharge motor was adjusted based on the real-time flow feedback, so as to realize the precise control of the fertilizer discharge amount.

**Figure 3:**
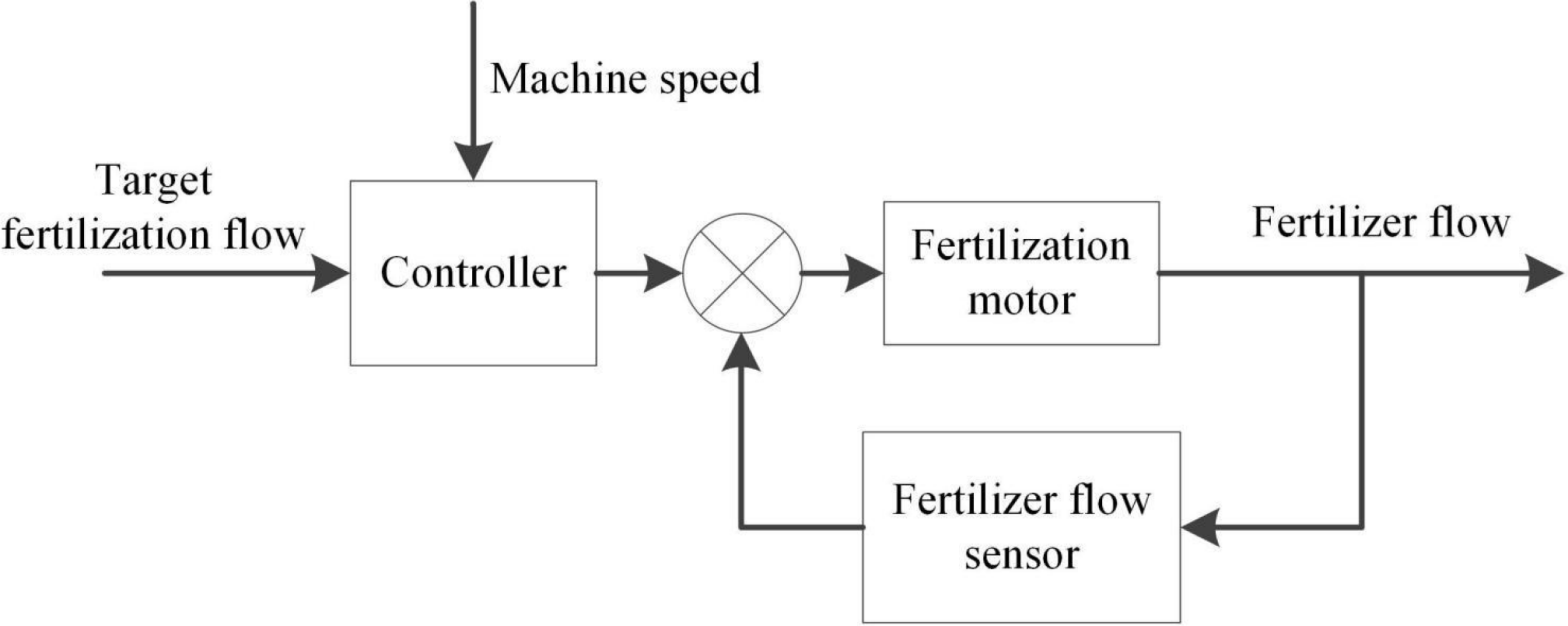
Block diagram of fertilizer flow control system.

The control model of the fertilizer flow control system takes the real-time vehicle speed collected by the Hall speed sensor as the input. After calculation, the controller sends the electrical signal to the fertilization mechanism, which adjusts the speed to control the fertilizer flow. Finally, the output of the system was the fertilizer flow. As shown in **Figure 3**, the fertilizer flow was fed back to the controller through the fertilizer flow detection module, and the closed-loop negative feedback control was carried out through the controller.

According to the required amount of fertilizer applied per unit area, the fertilizer flow can be controlled in real time by the operating width and forward speed of the fertilizer machine.


(7) $$Q(t)=A_{f}W\cdot v\left(t\right)$$

where, *Q*(t) is used to represent the real-time controlled fertilizer flow, g/s; *A_f_* is used to represent the amount of fertilizer applied per unit area, kg/m^2^; *W* is used to represent the operating width of the fertilizer applicator, m; *v*(t) is used to represent the real-time forward speed of the machine, m/s.

As can be seen from **Figure 3**, the control system can feedback adjust the speed of the fertilizer motor on the basis of obtaining the fertilizer flow rate to realize the real-time control of the fertilizer flow rate.

The sensor is turned on and allowed to run without load for a period of time. The micro-controller STM32F 103C8T6 averages the 30 capacitance values collected by the capacitance digital conversion chip PCAP01 (ACAM company, Germany) and was recorded as the initial capacitance value *C*_0_. When fertilizer passes through the fertilizer guide pipe, the sensor capacitance value will change. STM32F 103C8T6 was used to record and calculate the accumulated difference between the real-time capacitance value and the initial capacitance value within 1s, and calculate the fertilizer quality passing through the fertilizer guide pipe within 1s according to the relationship between capacitance value and fertilizer quality (6), which was recorded as fertilizer flow. CAN information was sent by the built-in CAN bus controller of STM32F 103C8T6 through SN65HVD230 transceiver, so as to realize the collection of real-time flow value of fertilizer.

When the system was in working condition, the difference between the target fertilizer flow and the real-time fertilizer flow was used as the control deviation.


(8) $$e=Q_{a}-Q$$

where, *e* is used to represent the control deviation, g/s; *Q_a_* is used to represent the target fertilizer flow, g/s.

The discrete PID control expression is:


(9) $$\text{u}\left(t\right)=K_{P}e\left(t\right)+K_{I}\sum_{n=0}^{t}e\left(n\right)+K_{D}\left(e\left(t\right)-e\left(t-1\right)\right)$$

where, u(*t*) is used to represent the control quantity; *K_p_*,*K_I_*,*K_D_* were used to represent the proportional, integral and differential constants, respectively; *e*(*t*) is used to represent the control deviation value at time *t*.

The control system tuner in Simulink was used to optimize the controller parameters according to different error values. The specific process of PID controller parameter adjustment is to set a target fertilizer flow value in advance and record the deviation of fertilizer flow value. When the deviation of fertilizer flow is very small. PID control was used to take the value of *K_I_* larger and the value of *K_p_* to the minimum. This can eliminate the static error in time and improve the control precision of the control system. When the deviation of fertilizer flow is small. PID control was used to take the value of *K_I_* smaller, take the value of *K_p_* smaller. This can prevent system overshoot in time. When the deviation of fertilizer flow is large. PD control was used to set the value of *K_I_* to 0 and the value of *K_p_* to the maximum. This prevents overshoot and allows the system to respond quickly to the target fertilizer flow rate. After optimization, *K*_p_=1.474,*K_I_* =0.0606 and *K_D_* =1.317.

#### 3.2.2 Design and testing of control system

**Figure 4:**
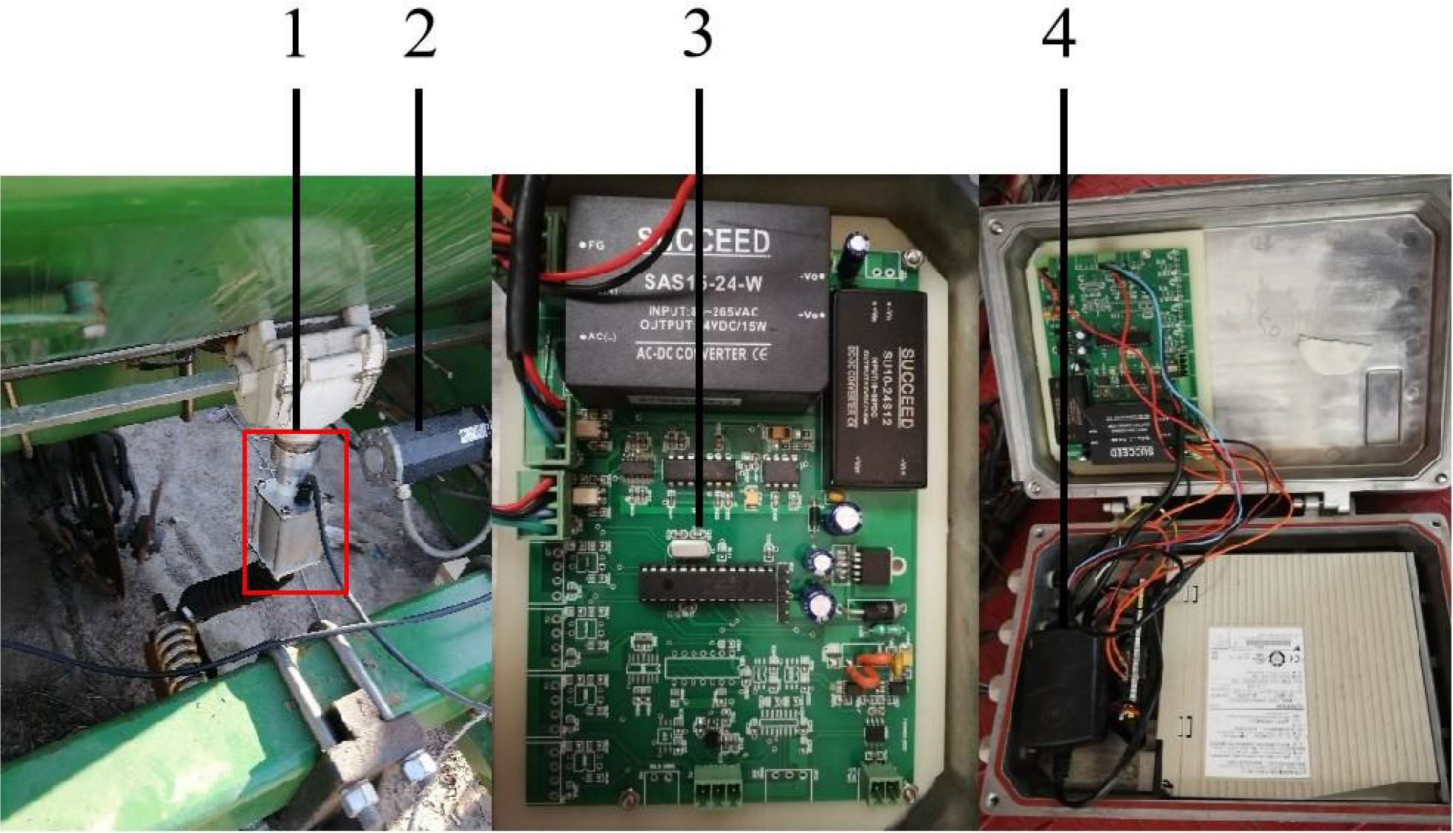
Precision fertilization control system equipment with fertilization flow sensor. 1.Capacitive flow sensor 2. Fertilization motor 3. Controller 4. Control box.

Based on closed-loop control technology of fertilizer flow feedback, the capacitance fertilizer flow sensor was installed on the fertilizer discharge pipe to detect the fertilizer flow information in real-time, and it was used as a feedback input. The PID control algorithm was used to control the system based on the established fertilizer application rate, the information of fertilizer flow and forward speed detected in real-time. The motor speed was controlled, so as to realize the closed-loop control of the fertilization amount. The developed corn precision fertilization control system is mainly consists of on-board terminals, electric drive fertilization units, and fertilization flow sensors, as shown in **Figure 4**.

**Figure 5:**
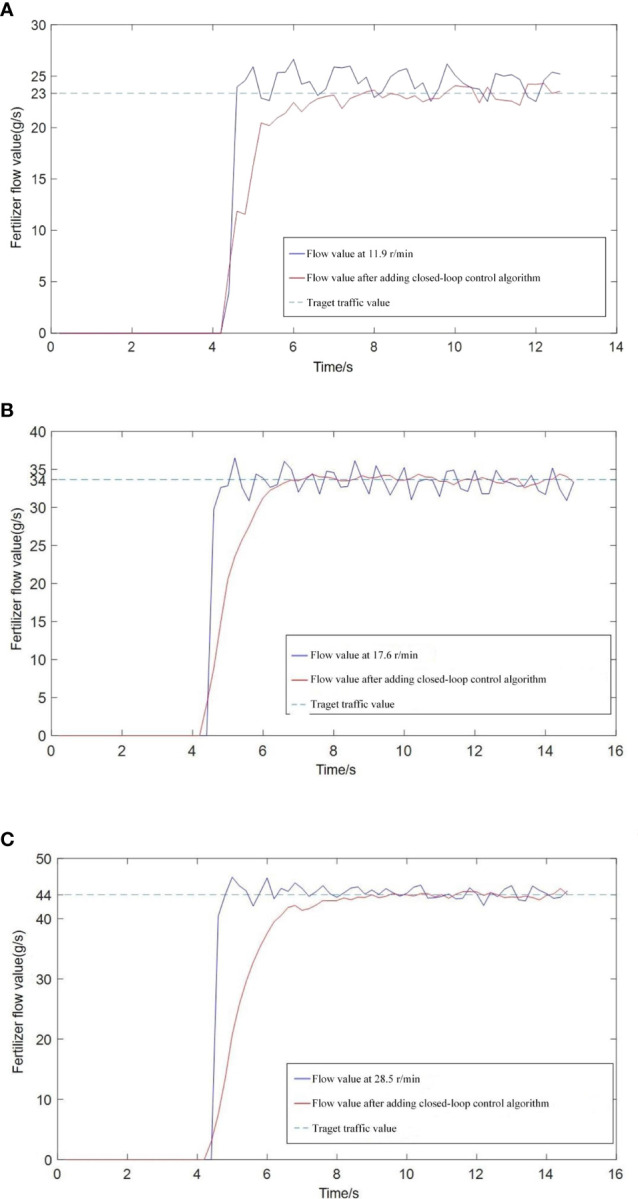
Response curve of fertilization rate under different set fertilization flow rate. **(A)** Set the fertilizer flow rate to 23 g/s; **(B)** Set the fertilizer flow rate to 33 g/s; **(C)** Set the fertilizer flow rate to 44 g/s.

The precision fertilization control system was tested and validated. Three different expected fertilization flow were set respectively, and the adjustment of fertilization amount under two different control modes of PID closed-loop control and speed adjustment was compared, as shown in **Figure 5**.

It can be seen from the **Figure 5** that using the traditional speed adjustment method, the amount of fertilizer discharge will continue to fluctuate after reaching the set flow rate. The main reason is that the fertilizer distributor has a grooved wheel structure. The occurrence of pulsation leads to uneven fertilization, and the application of PID control can directly control the fertilization flow, which reduces the influence of pulsation on the fertilization flow. In addition, after using the PID control method of flow feedback, the steady-state error of fertilizer discharge can be controlled within 2%, while the steady-state error of the simple speed adjustment method was about 4%. Therefore, the use of the fertilization flow control method based on flow feedback can effectively improve the control precision of fertilization amount.

**Figure 6:**
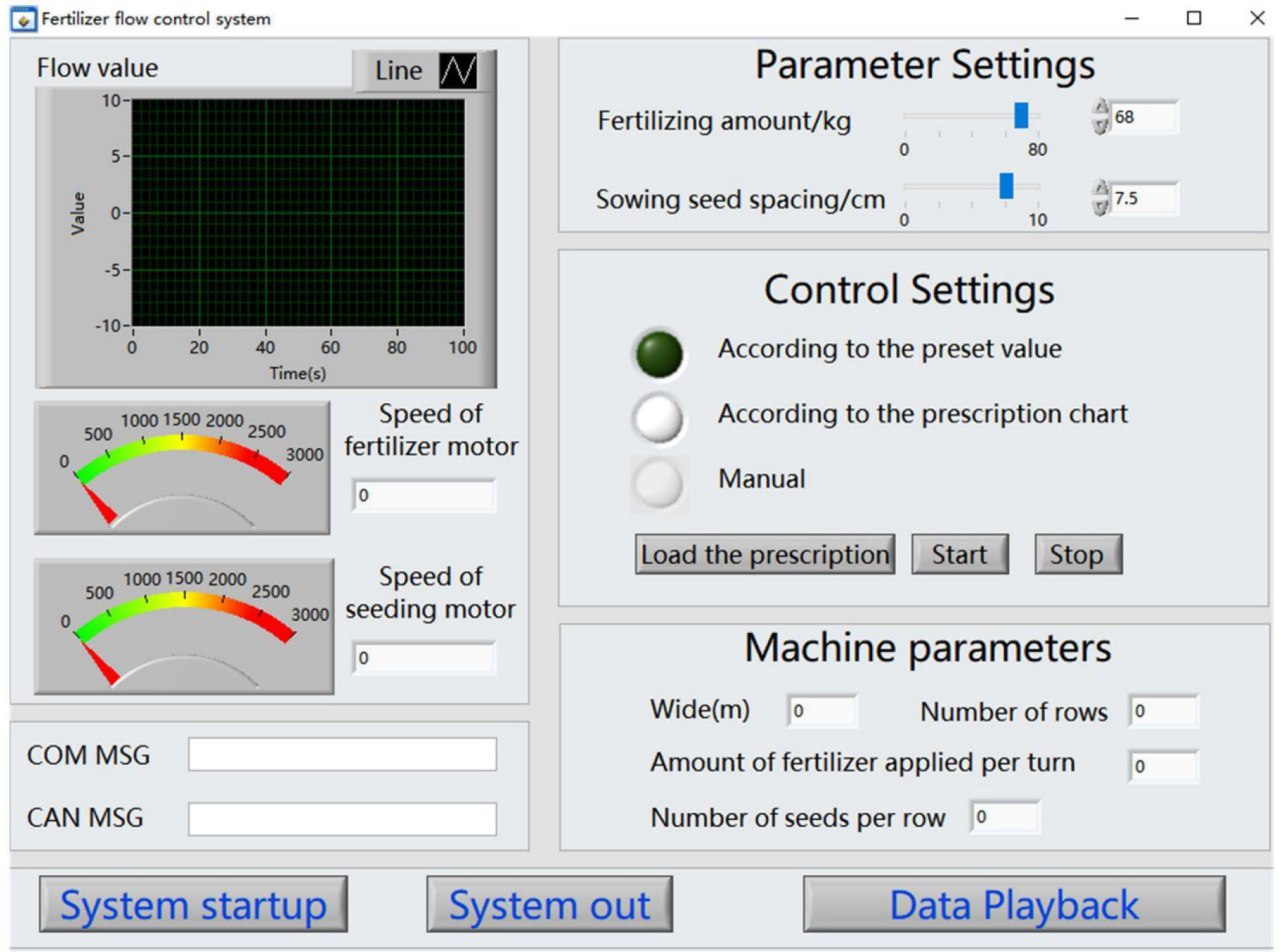
The main interface of the monitoring system.

The vehicle terminal adopts IEI Ikarp type vehicle computer, adopts Labwindows CVI2012 software environment for vehicle terminal software development, and uses Windows 7 operating system as the system software running platform. The interface is as shown in **Figure 6**. The software adopts the principle of modular and structured design, which mainly including data acquisition module, data storage module, data display module and parameter configuration module. It mainly realizes the acquisition and display of seeder running state information, real-time acquisition of GPS positioning information and variable control of sowing and fertilization. And real-time storage and processing of system data.

Data acquisition and control is the key components of the variable measurement and control system software for corn planters. This part mainly completes the collection of various data information such as sowing quality, seed fertilizer box material level, GPS and other information. There are two communication modes between the external devices of the system and the interactive terminal: RS 232 serial communication and CAN bus communication. Among them, the sowing quality information and the vehicle speed information communicate with the interactive terminal through the CAN bus. The measurement and control system determines the expected speed of seeding and fertilization according to the loaded prescription information, the current GPS information of the vehicle, the parameters of the implement and so on. And sends it to the control unit through the CAN bus to control the fertilization and seeding motor to run at the desired speed.

## 4 Results of field test

### 4.1 Test conditions

In order to test the fertilization control performance of the control system at different operating speeds and compare it with the performance of traditional mechanical, a field test was conducted in Shengfa Agricultural Machinery Cooperative, Jiangjiazhuang Village, Jimo District, Qingdao City, Shandong Province in September 2019. This test field is 180 meters long and 30 meters wide. The soil condition is no-till land just after harvesting summer corn, and the stubble on the surface is clearly visible. The system in this study was based on the design of one row of seeding unit of Zhongnong Machinery 2BJ-470B 4-row corn no-tillage precision seeder. It has been installed and debugged, and the adjustment performance of one row of mechanical sowing units of the original planter has been compared.

According to the agronomic requirements of this area, before the test, the sowing fertilization depth of the two rows of sowing monomers under the two adjustment methods was adjusted to 50 mm through the sowing depth adjustment handle. The test plot was divided into 3 plots, and the tests were conducted at the speed of 4, 6, 8 and 10 km/h, respectively. The Beidou satellite speed measurement module was installed on the tractor. The speed of the test group was kept within the range of 3~5 km/h, 5~7 km/h, 7~9 km/h, and 9~11 km/h, respectively.

Before starting the tractor, adjust the working parameters of the mechanically adjustable sowing device according to the user manual.

**Figure 7:**
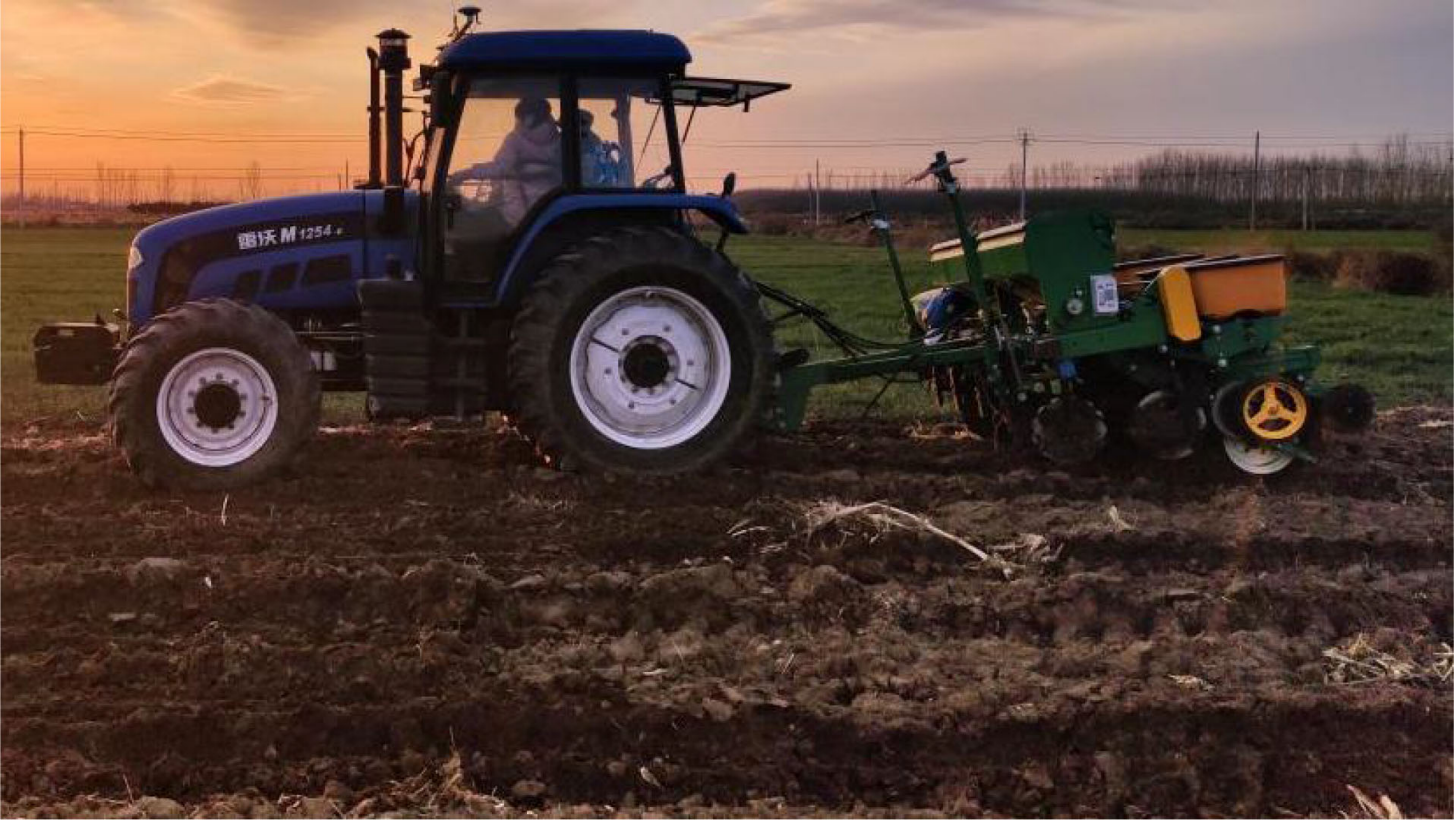
Field test.

The field test is shown in **Figure 7**. The monomers corresponding to Nos. 4 and 1 were the monomers used in the field test.

### 4.2 Consistency test of electric drive for fertilizer removal

Under the forward speeds of 4, 6, 8, and 10 km/h, respectively, the fertilizers discharged from the 4 fertilizer discharges were connected to the fertilizer receiving box. The fertilizer used in the experiment was “Dili Changxin” stabilized compound fertilizer. An electronic balance (SL-4001 electronic balance, indicating value accuracy ±0.1g) was used to weigh the amount of fertilizer applied in each row, and calculate the coefficient of variation of fertilization. The results are shown in **Table 3**.

**Table 3:** Consistency test of electric drive for fertilizer removal.

Number	Forward speed(km/h)	Sowing unit 1 fertilizer rate (g)	Sowing unit 2 fertilizer rate (g)	Sowing unit 3 fertilizer rate (g)	Sowing unit 4 fertilizer rate (g)	Average amount of fertilizer(g)	Standard deviation	Variation Coefficient of Fertilization (%)
1	4	98.2	96.4	96.4	101.4	98.1	2.357	2.4
2	4	90.9	90.9	91.3	92.5	91.4	0.757	0.83
3	4	91.6	91.6	90.4	90.6	91.05	0.64	0.7
4	6	136.5	137.5	136.6	138.5	137.275	0.932	0.68
5	6	140.4	140.2	135.8	136.5	138.225	2.414	1.75
6	6	134.5	135.8	140.2	140.2	137.675	2.964	2.15
7	8	183.6	182	181	183	182.4	1.143	0.63
8	8	185.0	180.6	181.6	186.3	183.375	2.711	1.48
9	8	180.5	180.0	185.3	177.5	180.825	3.259	1.8
10	10	229	227	225	230	227.75	2.217	0.97
11	10	228.5	235.6	228.3	224.5	229.225	4.631	2.02
12	10	225.6	240.6	220.5	230.6	229.325	8.573	3.74

It can be seen from **Table 3** that the system electric drive fertilizer discharge consistency was good. The average standard deviation of fertilization was 2.7165. The maximum standard deviation of fertilization was 8.573. And the maximum and average variation coefficient of fertilization were 3.74%, 1.6%, respectively. China National standard stipulates (GB/T20865-2007) that the coefficient of variation of corn fertilization planter should be less than or equal to 7.8%. Therefore, the fertilizer discharge performance of the corn fertilization planter in this study can meet the actual production needs.

### 4.3 Performance test of electric drive fertilizer control system based on flow feedback

The performance test of the precision fertilization control system was further carried out. The fertilization rate were set at 30, 40, and 50 kg/mu, respectively. And the results of fertilization rate control were recorded, as shown in **Table 4**.

**Table 4:** Test data of fertilization control.

Set the amount of fertilizer (kg/mu)	Test serial number	Converted discharge (g)	Actual amount of fertilizer (g)	Control precision (%)
30	1	2462	2475	99.47
	2	2462	2326	94.46
	3	2462	2460	99.88
40	1	3283	3532	92.96
	2	3283	3159	96.22
	3	3283	3279	99.87
50	1	4104	4178	98.21
	2	4104	4271	96.07
	3	4104	3975	96.85
Average value				97.11

It can be seen from **Table 4** that the optimized precision fertilization control system can realize the on-demand use of the fertilization amount. And its variable control accuracy was more than 97%. When the fertilization rate were set at 30, 40, and 50 kg/mu, the standard deviation of fertilization control precision were 3.018, 3.456 and 1.083, respectively. Which can meet the actual production needs. The test of the roller hole fertilizer application between corn rows studied by Wan et al. (2020). showed that the deviation of fertilizer output was 11.2%. An et al. (2017) used electro-hydraulic proportional control technology to control the fertilizer discharge shaft of corn fertilizer planter, and the error of fertilizer was less than 3%. The fertilization control accuracy of this study is similar to or slightly better than those of related scholars. Therefore, the optimized precision fertilization control system in this study can meet the actual production needs.

Before the test, the sowing grain spacing were set to 20 cm, 25 cm, and 30 cm in the system interface, respectively. The tractor pulled the seeder forward 100 m, and the precision seeding controller was used to control the seed meter for sowing operations. In order to facilitate the measurement of the seed spacing, the covering soil was suppressed before the operation. The wheel was raised so that the seeds fall in the groove and are not covered. After the test, pinch the head to the tail, select the middle 50 m, manually measure the seed spacing and compare it with the set grain spacing. The results are shown in **Table 5**.

**Table 5:** Test data of precision seeding control system.

Theoretical grain distance/cm	Seeding unit number	Actual grain distance (cm)	Control precision (%)
20	1	19.46	0.97
	2	19.79	0.99
25	1	24.73	0.99
	2	24.99	1.00
30	1	28.90	0.96
	2	29.29	0.98
Average value			0.98

It can be seen from **Table 5** that the control accuracy of the grain spacing control by electric drive seed metering was 98%. When the sowing grain spacing were set to 20 cm, 25 cm, and 30 cm, the standard deviation of grain spacing control accuracy were 0.014, 0.007 and 0.014, respectively. The test of the corn high speed air suction seed metering device studied by Liu et al. (2022). showed that the grain distance qualification index was greater than 94.6%, which was higher than the national standard of China. Therefore, the grain spacing control accuracy of the electric drive seed metering control system in this study can meet the actual production needs.

## 5 Conclusion

(1) Aiming at the problems for excessive fertilizer use and poor uniformity of fertilizer discharge in corn fertilizer planter. A set of accurate perception and control system applied to corn fertilization planter was studied. By installing a capacitance fertilization flow sensor on the fertilization pipe, the fertilizer flow information is detected in real time and used as a feedback input. The control system controls the speed of the fertilizer discharge motor based on the PID control method according to the set fertilizer amount and the real-time detection of fertilizer flow and forward speed, so as to realize the closed-loop control of the fertilizer amount. As the temperature rises from room temperature to 55°C, the differential capacitance change rate of the sensor was less than 3%. The detection accuracy of the fertilization amount capacitance sensor for different fertilizer quality was greater than 96%. The steady-state error of fertilizer discharge was less than 2%.

(2) Design experiments to test the performance of the variable measurement and control system for corn sowing and fertilization. According to the experimental data, the electric drive fertilization system has good consistency, and the maximum and average variation coefficient of fertilization were 3.74%, 1.6%, respectively, The variable control accuracy was more than 97%. When the fertilization rate were set at 30, 40, and 50 kg/mu, the standard deviation of fertilization control precision were 3.018, 3.456 and 1.083, respectively. The grain distance control accuracy of the electric drive seed metering control system was 98%. When the sowing grain spacing were set to 20 cm, 25 cm, and 30 cm, the standard deviation of grain spacing control accuracy were 0.014, 0.007 and 0.014, respectively. And the comprehensive performance can meet the actual production needs.

## Data availability statement

The raw data supporting the conclusions of this article will be made available by the authors, without undue reservation.

## Author contributions

Conceptualization, BW, HW, LZ and HM. Methodology, BW, LZ and HM. Formal analysis, BW and LZ. Data curation, BW, HW and LZ. Writing—original draft preparation, BW, YW, LZ and HM. Writing—review and editing, BW, YW, LZ and HM. All authors contributed to the article and approved the submitted version.

## Funding

This research was funded by National Bulk Vegetable Industry Technical System Post Expert (CARS-23-D03) and The National Natural Science Foundation of China (32071905).

## Conflict of interest

The authors declare that the research was conducted in the absence of any commercial or financial relationships that could be construed as a potential conflict of interest.

## Publisher’s note

All claims expressed in this article are solely those of the authors and do not necessarily represent those of their affiliated organizations, or those of the publisher, the editors and the reviewers. Any product that may be evaluated in this article, or claim that may be made by its manufacturer, is not guaranteed or endorsed by the publisher.

## References

[B1] An X. F. Fu W. Q. Wang P. Wei X. L. Li L. W. Meng Z. J. (2019). Development of variable rate fertilization control system based on matching fertilizer line and seed line of wheat. Trans. Chin. Soc. Agric. Machinery. 50 (S1), 96–101. doi: 10.6041/j.issn.1000-1298.2019.S0.016

[B2] An X. F. Fu W. Q. Wei X. L. Cong Y. Wang P. (2017). Evaluation of four-element variable rate application of fertilization based on maps. Trans. Chin. Soc. Agric. Machinery. 48 (S0), 66–70. doi: 10.6041/j.issn.1000-1298.2017.S0.011

[B3] Cai G. H. Li H. Li H. W. Wang Q. J. Ni J. L. (2011). Design of test-bed for automatic depth of furrow opening control system based on ATmega128 single chip microcomputer. Trans. CSAE. 27 (10), 11–16. doi: 10.3969/j.issn.1002-6819.2011.10.002

[B4] Chen J. C. Zhang H. Pan F. Du M. J. Ji C. (2022). Control system of a motor-driven precision no-tillage maize planter based on the CANopen protocol. Agriculture-basel 12 (7), 932. doi: 10.3390/agriculture12070932

[B5] Darwin B. Dharmaraj P. Prince S. Popescu D. E. Hemanth D. J. (2021). Recognition of Bloom/Yield in crop images using deep learning models for smart agriculture: A review. Agronomy-Basel 11 (4), 646. doi: 10.3390/agronomy11040646

[B6] Ding Y. Q. Liu Z. Chen C. Liu H. L. Luo J. Yu H. F. (2021). Functional detection method of application rate based on principle of dynamic weighing. Trans. Chin. Soc. Agric. Machinery. 52 (10), 146–154. doi: 10.6041/j.issn.1000-1298.2021.10.015

[B7] Fu W. Q. Dong J. J. Mei H. B. Gao N. N. Lu C. Y. Zhang J. X. (2018). Design and test of maize seeding unit downforce control system. Trans. Chin. Soc. Agric. Machinery. 49 (6), 68–77. doi: 10.6041/j.issn.1000-1298.2018.06.008

[B8] Hakansson I. Arvidsson J. Keller T. Rydberg T. (2011). Effects of seedbed properties on crop emergence: 1. temporal effects of temperature and sowing depth in seedbeds with favourable properties. Acta Agriculturae Scandinavica Section B - Plant Soil Sci. 61 (5), 458–468. doi: 10.1080/09064710.2010.506446

[B9] He X. T. Ding Y. Q. Zhang D. X. Yang L. Cui T. Zhong X. J. (2019). Development of a variable-rate seeding control system for corn planters part II: Field performance. Comput. Electron. Agriculture. 162, 309–317. doi: 10.1016/j.compag.2019.04.010

[B10] Huang D. Y. Zhu L. T. Jia H. L. Yu T. T. (2015). Automatic control system of seeding depth based on piezoelectric film for no-till planter. Trans. Chin. Soc. Agric. Machinery. 46 (4), 1–8. doi: 10.6041/j.issn.1000-1298.2015.04.001

[B11] Karayel D. Özmerzi A. (2002). Effect of tillage methods on sowing uniformity of maize. Can. Biosyst. Eng. 44, 2–23.

[B12] Li Y. H. Meng P. X. Geng R. Y. He K. Meng F. H. Jiang M. (2016). Intelligent system for adjusting and controlling corn seeding depth. Trans. Chin. Soc. Agric. Machinery. 47 (S1), 62–68. doi: 10.6041/j.issn.1000-1298.2016.S0.010

[B13] Liu R. Liu Z. J. Liu L. J. Li Y. H. (2022). Design and experiment of corn high speed air suction seed metering device with disturbance assited seed-filling. Trans. Chin. Soc. Agric. Machinery. 53 (9), 50–59. doi: 10.6041/j.issn.1000-1298.2022.09.005

[B14] Liu L. K. Yi S. J. (2019). Design and experiment of seeding performance monitoring system for suction corn planter. Int. J. Agric. Biol. Eng. 12 (4), 97–103. doi: 10.25165/j.ijabe.20191204.4185

[B15] Nielse S. K. Munkholm L. J. Lamande M. Norremark M. Edwards G. T. C. Green O. (2018). Seed drill depth control system for precision seeding. Comput. Electron. Agriculture. 144, 174–180. doi: 10.1016/j.compag.2017.12.008

[B16] Poncet A. M. Fulton J. P. McDonald T. P. Knappenberger T. Shaw J. N. Bridges R. (2018). Effect of heterogeneous field conditions on corn seeding depth accuracy and uniformity. Appl. Eng. Agriculture. 34 (5), 819–830. doi: 10.13031/aea.12238

[B17] Wang K. R. Xie R. Z. Ming B. Hou P. Xue J. Li S. K. (2021). Review of combine harvester losses for maize and influencing factors. Int. J. Agric. Biol. Eng. 14 (1), 1–10. doi: 10.25165/j.ijabe.20211401.6034

[B18] Wan L. Xie D. B. Li Y. Chen L. Q. (2020). Design and experiment of roller hole fertilizer application between corn rows. Trans. Chin. Soc. Agric. Machinery. 51 (11), 64–73. doi: 10.6041/j.issn.1000-1298.2020.11.007

[B19] Wen L. P. Fan X. F. Liu Z. Zhang Y. (2014). The design and development of the precision planter sowing depth control system. Sensors Transducers. 162 (1), 53–58.

[B20] Yang L. Yan B. X. Zhang D. X. Zhang T. L. Wang Y. X. Cui T. (2016). Research progress on precision planting technology of maize. Trans. Chin. Soc. Agric. Machinery. 47 (11), 38–48. doi: 10.6041/j.issn.1000-1298.2016.11.006

[B21] Yuan Y. W. Bai H. J. Fang X. F. Wang D. C. Zhou L. M. Niu K. (2018). Research progress on maize seeding and its measurement and control technology. Trans. Chin. Soc. Agric. Machinery. 49 (9), 1–18. doi: 10.6041/j.issn.1000-1298.2018.09.001

[B22] Yuan J. Liu C. L. Li Y. M. Zeng Q. B. Zha X. F. (2010). Gaussian Processes based bivariate control parameters optimization of variable-rate granular fertilizer applicator. Comput. Electron. Agriculture. 70 (1), 33–41. doi: 10.1016/j.compag.2009.08.009

[B23] Zhao Z. Li Y. M. Chen J. Xu L. Z. (2010). Numerical analysis and laboratory testing of seed spacing uniformity performance for vacuum-cylinder precision seeder. Biosyst. Eng. 106 (4), 344–351. doi: 10.1016/j.biosystemseng.2010.02.012

[B24] Zhao J. H. Liu L. J. Yang X. J. Liu Z. J. Tang J. X. (2015). Design and laboratory test of control system for depth of furrow opening. Trans. CSAE. 31 (6), 35–41. doi: 10.3969/j.issn.1002-6819.2015.06.005

